# A Comprehensive Study of the Mechanical and Durability Properties of High-Performance Concrete Materials for Grouting Underwater Foundations of Offshore Wind Turbines

**DOI:** 10.3390/ma14205968

**Published:** 2021-10-11

**Authors:** Wen-Ten Kuo, Zheng-Yun Zhuang

**Affiliations:** Department of Civil Engineering, National Kaohsiung University of Science and Technology, Kaohsiung City 807, Taiwan; wtkuo@nkust.edu.tw

**Keywords:** high-performance concrete (HPC), satisfied solution for proportioning, underwater foundations, durability, rapid chloride permeability test (RCPT), compressive strength, data analytics, cross-categorical inter-parametric relationships

## Abstract

With the increasing importance of offshore wind turbines, a critical issue in their construction is the high-performance concrete (HPC) used for grouting underwater foundations, as such materials must be better able to withstand the extremes of the surrounding natural environment. This study produced and tested 12 concrete sample types by varying the water/binder ratio (0.28 and 0.30), the replacement ratios for fly ash (0%, 10%, and 20%) and silica fume (0% and 10%), as substitutes for cement, with ground granulated blast-furnace slag at a fixed proportion of 30%. The workability of fresh HPC is discussed with setting time, slump, and V-funnel flow properties. The hardened mechanical properties of the samples were tested at 1, 7, 28, 56, and 91 days, and durability tests were performed at 28, 56, and 91 days. Our results show that both fly ash (at 20%) and silica fume (at 10%) are required for effective filling of interstices and better pozzolanic reactions over time to produce HPC that is durable enough to withstand acid sulfate and chloride ion attacks, and we recommend this admixture for the best proportioning of HPC suitable for constructing offshore wind turbine foundations under the harsh underwater conditions of the Taiwan Bank. We established a model to predict a durability parameter (i.e., chloride permeability) of a sample using another mechanical property (i.e., compressive strength), or vice versa, using the observable relationship between them. This concept can be generalized to other pairs of parameters and across different parametric categories, and the regression model will make future experiments less laborious and time-consuming.

## 1. Introduction

The critical role of wind power for renewable energy (RE) planning has been addressed in many studies [1,2,3]. In general, wind energy is captured either on land or offshore before the electricity produced is converted and transmitted through the grid [4]. Wind energy is mainly exploited on land at inland locations, such as the edge of deserts [5,6] or on the seashore [7], where constant wind speeds able to drive turbines are available all year. However, because there are also many such high-quality locations on (above) sea with very low land-acquisition costs, offshore wind resources have attracted increasing attention in the past decade [8]. This option has already been included in some major strategic plans or energy policies and integrated with other RE sources, e.g., even hydrogen energy [9], for sustainable development. Appendix A.1 provides the extensive discussion.

The island of Taiwan, explored in the 16th century by the Portuguese who named it Ilha Formosa, is completely surrounded by the sea, and this natural advantage will determine its destiny in the upcoming years to exploit offshore wind energy (in addition to onshore wind energy). In particular, the west coast of the island faces the Taiwan Strait, a continental shelf where the sea’s topography is shallow, and several high-quality locations in the strait, e.g., the Taiwan Bank, for installing offshore wind turbines have already been identified [10].

However, a key engineering problem before the installation of wind turbines is the building of their undersea foundations. In civil engineering, the successful construction of these foundations involves not only knowledge of structuring, but also related knowledge to ensure that the materials selected, e.g., concrete and rebar or steel bar, can withstand the extreme conditions in the surrounding natural environment [11]. For example, it is well understood that epoxy-coated reinforced steel bars can be used as a replacement for normal steel bars to greatly extend the age of the structure [12,13,14]. Previous studies have shown that the chloride and sulfate ions in seawater have a strongly corrosive effect on concrete materials. The presence of these ions is determined by the climatic and geological features of the area (see Appendix A.2 for more detail). Therefore, the use of high-performance concrete (HPC) is required to ensure that constructions are more durable under such extremely harsh environmental conditions [15,16,17].

In construction engineering, HPC is mostly used in roadwork or for the structural engineering of bridges. Although the requirements for concrete used in high load-bearing road structures and port buildings should be similar, many recent uses of HPC have been inspired by either advanced marine engineering projects or the construction of offshore wind turbines [18]. Irrespective of how HPC is used in the marine environment, the challenges always involve substances that are harmful but common in the sea, i.e., chloride and sulfate ions [19].

The undersea structures are the foundations for the wind turbines that will be established in the Taiwan Bank. Since at least one such structure could easily be affected by natural extremes, higher durability and better mechanical properties of the material (either fresh or hardened) used must be ensured, depending on the surrounding environment. Therefore, the aim of our experiments is to identify the best (or better) mixture of the HPC material used in this context. We also argue that replacing the cement in the HPC material with fly ash (FA), silica fume (SF), and slag may ensure the quality of the mixture. Accordingly, we review the relevant studies in greater detail below.

We performed two tests to evaluate the fresh mechanical properties, three tests to determine the hardened mechanical properties, and two tests to evaluate the durability of each of the 12 types of samples produced using two water/binder (or water/cement; W/B) ratios of 0.28 and 0.30 and different replacement ratios of FA (0%, 10%, and 20%) and SF (0% and 10%) to substitute for cement; the replacement proportion of slag was controlled intentionally at 30% in the experimental design. Therefore, the most suitable HPC material for construction could be judged by comparing the test results for these experimental conditions. Note that the fixed replacement proportion of slag applied in the tests references those well accepted by practitioners for making their concrete mixes in Taiwan.

We also argue that additional data analyses may help to identify a suitable model to establish the link between the different categories of variables. Therefore, a highlight of this study is the employment of methodology from the data analytics field to model the relationship between compressive strength (an index of hardened mechanical properties) and chloride ion permeability (an index of durability) based on data for all the tested samples. We used K-means clustering for unsupervised machine learning and simple linear regression (SLR), with several formal trials, to identify the relationship between these two indices using a method for dimensional alternation. Validating the effectiveness of the established model allows it to be used to predict an index by using another. Therefore, in addition to the valuable knowledge gained from the main experimental processes, the model may save time and effort spent on future experiments. Consequently, one test item can be omitted, and results are predicted from those obtained for another test item across the parametric categories. A review of the uses of FA, slag, and SF in the proportioning of the constituents of HPC is given below.

Ali et al. (2019) [20] found that concrete mixed with FA (at 20% and 40%) may show higher strength and fewer interstices, while suffering less from permeation of chloride ions compared to a control. Bharatkumar et al. (2005) [21] showed that when the slag component in concrete samples is replaced by FA, the durability of the samples also increased. These studies confirm the role of FA in the proportioning of HPC mixtures.

Cheng et al. (2005) [22] replaced the cement in a concrete sample with ground granulated blast furnace slag (GGBFS) and conducted a rapid chloride ion permeability test (RCPT). They found that the sample with GGBFS became less permeable to chloride ions, with a reduced rate of permeability (distance over time) and reduced accumulated electric charge passed, with an increasing GGBFS ratio. Recently, Kim et al. (2021) [19] also found that the replacement of cement with GGBFS made HPC more durable in seawater environments and provided the results of a long-term evaluation to demonstrate the material’s higher performance when used to construct foundations for offshore wind turbines. These studies confirm the role of slag in the proportioning of HPC mixtures.

Moffatt and Thomas (2018) [23] demonstrated the long-lasting durability of HPC samples with SF and FA in the mixture when exposed to severe marine conditions for 25 years. A positive effect was also shown for chloride ion permeability; in the samples with SF and FA, the penetration of chloride ions was shallower than in the samples without these additions (40 mm vs. 90 mm). In other words, the combined use of SF and FA makes HPC more impermeable to chloride ions, with a possible reduction in corrosive or erosive effects. In a more recent study, Mustapha et al. (2021) [24] fixed the replacement ratio of SF (substituting for cement) at 10% and tested a range of replacement ratios of FA (0% to 75%). Despite the pozzolanic reaction becoming slower with higher additions of FA, the strength of the material increased to a large extent over time, i.e., its compressive strength became 87.06 MPa by day 28 with 50% of the cement in the mix replaced by FA and 10% by SF. Satish et al. (2017) [25] made 135 cubic and 90 cylindrical HPC samples and showed that the inclusion of SF in the mixture provided greater strength than the inclusion of FA. The strength of the material always increased as the substitution ratio of SF increased, and the magnitude of the strength increase was greater than that with increased FA substitution. However, FA remained an essential component because of the machinability it afforded to the HPC material. These studies demonstrate the role of SF in the HPC mixture depending on the context, but it was rare for it be used alone, i.e., it was usually used together with FA.

Other recent studies also show the positive outcomes of using both SF and FA in concrete mixtures. Akhnoukh and Elia (2019) [26] aimed to design an economical HPC material and argued that binding materials such as SF and FA (specifically, C-type FA) can be added to obtain the desired properties of HPC. Their experimental results showed that samples could be produced with a compressive strength of 105 MPa at 28 days and 70 MPa at only 24 h. The results of the accelerated mortar bar test further demonstrated that the concrete with supplementary cementing material was not vulnerable to alkali–silica reactions. Therefore, the best HPC material to be produced was applied in a construction project.

In addition to the above studies directly related to our work, other relevant research efforts are worth reviewing in brief. Khotbehsara et al. (2015) [27] mixed nano-copper oxide (NC; 1–4%) and FA (20–30%) in self-compacting concrete and tested the samples’ mechanical properties. They tested the slump flow and the time elapsed to flow through a V-shaped funnel (for fresh mechanical properties) and the compressive strength of samples at 7, 28, and 91 days and conducted RCPT tests. The sample with 3% NC and 25% FA yielded the highest improvement in mechanical properties. In general, with increased FA, the sample not only became more impermeable to chloride ions and more resistant to the conduction of electricity, but also possessed greater endurance and strength due to the denser material packing in the interstices. A recent study by Sohail et al. (2021) [28] compared the durability of HPC and ultra-HPC (UHPC) against normal-strength concrete (NSC). Several features of the three types of concrete, all produced without any heat treatment, were compared, with observations made using scanning electron microscopy and X-ray diffraction. A major (and interesting) finding of the study is that the coefficient of diffusion of UHPC was lower than that of NSC by three observable levels; this could postpone the corrosion of the reinforcing steel inside the concrete. Lastly, de Medeiros-Junior et al. (2015) [29] assessed the effects of chloride penetration on an offshore concrete platform between 2000 and 2005. They studied how chlorides penetrated the concrete material and how they were distributed at three locations on the platform: one location exposed to sea air, one exposed to the tides, and another exposed to seawater spray. They found that the degree of chloride penetration was proportional to the duration of exposure and that weather factors (e.g., windy/not windy or humid/dry) affected the distribution of chlorides.

This review establishes the roles of FA, slag, and SF, which are the main additions used for proportioning HPC in this study. However, from our review of the literature, we found that studies identifying relationships between the different parameters of concrete samples are relatively rare. Therefore, in addition to the seven fresh or hardened mechanical properties or the durability measures tested for the 12 sample types to understand the best combination of materials to produce the optimal HPC for constructing offshore wind turbine foundations, we develop a model to clarify the link between compressive strength and charge passed, so that it can be used to predict an unknown parameter using a known one. As this model predicts a hardened mechanical property using a durability measure (and vice versa), and it has been proven to work effectively across these two parameters, the concept can be generalized for possible future trials to establish predictive models with other pairs of concrete properties. 

This paper is organized as follows. Section 2 details the experimental design. Section 3 presents the results of each test for each proportioned sample. Section 4 summarizes the main results, explores insights in depth for engineering practice, establishes the predictive model, and identifies unforeseen cross-category relationships through analytical methods. Section 5 presents the conclusion of this study.

## 2. Experimental Design

### 2.1. The Test Methods

To test the initial mechanical properties of the samples, the slump flow (cm) and the time elapsed for a sample to flow through the V-shaped funnel (s) were measured. These two tests were performed for fresh properties of the concrete samples. To measure the slump flow, we followed the methods in ASTM-C143 (ASTM International, 2020) [30]. To test the time taken to flow through the V-shaped funnel, we followed the standard regulated in ASTM-C1362 (ASTM International, 2002) [31].

The samples’ other non-initial mechanical properties, compressive strength (MPa), ultrasound pulse velocity (m/s), and surface (sheet) resistivity (kΩ-cm), were measured after certain periods of time. These three tests were performed for the hardened mechanical properties of the concrete samples. The parameters were measured for each sample at 1, 7, 28, 56, and 91 days during the aging process. Therefore, these measures could be compared between the different (aging) stages of the sample under different proportioning conditions. We also followed the ASTM standards C39 (ASTM International, 2021) [32], C597 (ASTM International, 2016) [33], and C876 (ASTM International, 2015) [34] to test the compressive strength, ultrasound pulse velocity, and surface resistivity, respectively, of each sample on those specified days. Note that the ultrasound pulse velocity (USPV) measure based on C597 (entitled “Standard Test Method for Pulse Velocity Through Concrete”) is negatively correlated with the interstices in the material; thus, it is positively correlated with the density of the material. The resistivity test uses a four-pole concrete resistivity meter to measure the resistivity of each sample.

The durability of these samples was the most interesting parameter to observe. Relevant experiments were performed to measure the ability of each sample to withstand harmful substances. These experiments also used two ASTM standards, ASTM C1012 (ASTM International, 2018) [35] and ASTM C1202 (ASTM International, 2019) [36], to expose concrete samples to sulfate and chloride ions and then to measure the sulfate resistance and permeability of chloride ions at 28, 56, or 91 days (because a shorter period would be meaningless for these measures). In these tests, sulfate resistance was measured in terms of the loss in weight of the sample (%). The greater the weight loss, the less the sample is able to resist sulfate ions, that is, the less durable it is. For testing chloride ion permeability, the electric charge (coulomb or C) for chloride ions, which could pass the sample successfully after acceleration, was measured when the ions were applied. The smaller the electric charge, the more impermeable to chloride ions the sample is, that is, the more durable it is. Further details of the methods used for the RCPT test are in the Chinese National Standards [37]. 

### 2.2. The Concrete Samples

Following from Section 1, for the purposes of our experiments, some additions were applied at different levels to produce concrete samples. Firstly, as the basic experimental condition for the material, two W/B ratios, 0.30 and 0.28, were used. This initially divided the samples into two main groups. The use of these two W/B ratios as the parameters was in accordance with the subject of this study—to explore the suitable proportions of the HPC used for underwater foundations of offshore wind turbines. Therefore, high strength, workability, and durability were required in this context. Secondly, the replacement ratios for FA (i.e., the ratio to replace the cementitious part with FA) were 0% (none), 10%, and 20%. Thirdly, the replacement ratios for SF were 0% and 10%. These treatments further partitioned each main sample group into six to produce 12 sample types, as mentioned previously, according to 12 different combinations of proportioning. Note that to control the experimental conditions, all samples were subject to a fixed replacement ratio of 30% of GGBFS and the addition of a superplasticizer at 1.5%.

To facilitate the relevant experiments and the presentation of results, these 12 sample types were coded as follows: W/B = 0.30 and W/B = 0.28 were used for the two W/B ratios; FA 0%, FA 10%, and FA 20% represented the three replacement ratios for FA; and SF 0% and SF 10% represented the two replacement ratios for SF. Table 1 shows the unique code and mixture portfolio of each sample.

**Figure 1:**
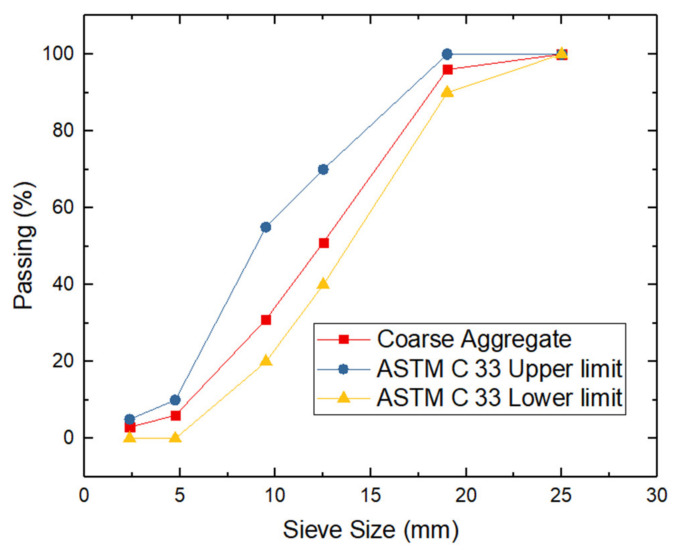
Grading curve of the coarse aggregate.

**Figure 2:**
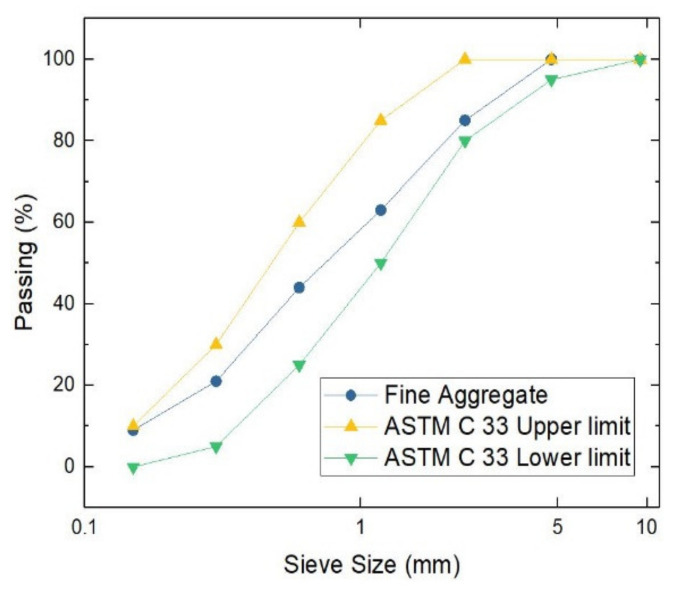
Grading curve of the fine aggregate.

The aggregates used, both fine and coarse, conformed to the standards regulated by ASTM C33. Table 2 shows a summary and Figure 1 and Figure 2 show the grading curve of the coarse and fine aggregate, respectively.

### 2.3. Binding Materials and the Superplasticizer

The binding materials used in this study, including Type I cement, GGBFS, FA, and SF, were tested for their basic parameters, including specific gravity and the fineness of powder (Table 3).

Table 4 provides supplementary information for the G-type superplasticizer admixture used at a fixed proportion.

## 3. Results

This section provides the main results for seven individual tests with three testing categories for the 12 concrete sample types (see Table 1). Each sample type was tested for two fresh properties, three hardened mechanical properties, and two durability properties. 

### 3.1. Fresh Properties

The fresh properties of concrete samples are parametric indices that can be used to measure a concrete material while it is still fresh (freshly made). In this study, we measured the slump flow (cm) of the sample and the time elapsed for the sample to flow through the V-shaped funnel (s). All 12 samples were tested for these two measures.

#### 3.1.1. Slump Flow

**Figure 3:**
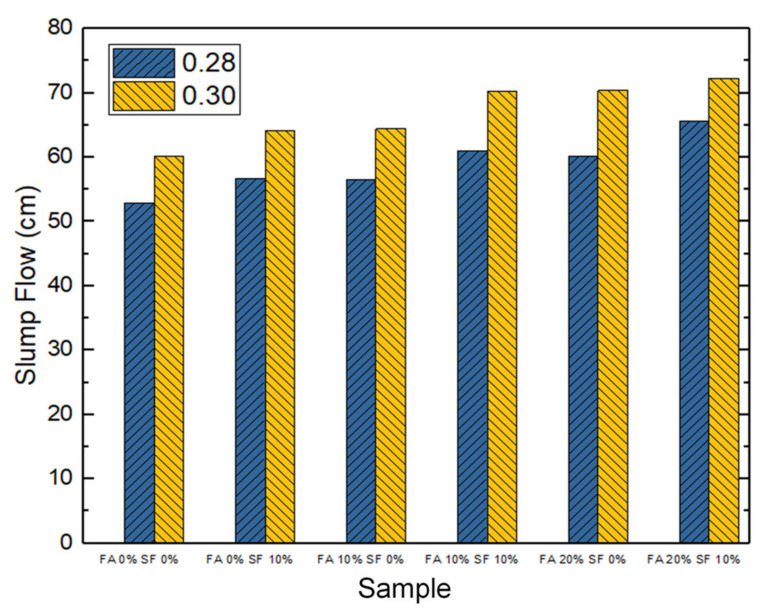
Slump flow data for the 12 HPC proportioning conditions (mixture combinations) with a water/binder (W/B) ratio of 0.28 or 0.30. Note: FA = fly ash; SF = silica fume.

A better slump flow usually implies that a concrete sample has better workability in practice. The slump flow of each sample was tested and measured. As can be seen in Figure 3, all slurries of the 12 concrete samples had the desired workability. In addition, a general trend observed was that as the cement content in the binding material decreased, the slump flow increased.

Further scrutiny revealed that the replacement of cement with both FA and SF improved slump flow. For example, for the samples with a W/B ratio of 0.28, the replacement of 10% of the cement with FA and 20% with SF improved slump flow by 24% (from 52.95 cm to 65.65 cm), compared to using neither material (i.e., FA = 0% and SF = 0%). In the case of samples with a W/B ratio of 0.30, the replacement of 20% of the cement with FA and 10% with SF also improved slump flow by 20% (from 60.25 cm to 72.3 cm) compared to using neither. In either case, the cement content was minimized (because of the substitution rates), and boule-shaped (pellets) granules in the FA and SF powders lubricated the ingredients in the mixture.

Moreover, the 30% fixed proportion of slag also facilitated the above effect. When replacing a part of the cement with the same weight of GGBFS, the volume occupied by the paste increases, i.e., the ratio of paste volume to aggregate volume increases, and a better lubricating effect can be obtained. In addition, compared with cement particles, GGBFS can form a reactive vitreous mixture due to high-temperature water quenching. As such, the surface does not absorb water and can increase free water. Therefore, being mixed with the same volume of water, the concrete with GGBFS has a slump greater than that of other concrete formulations without GGBFS. Thus, it was concluded that these characteristics improved the workability of the concrete mixture.

#### 3.1.2. Time Taken to Flow through the V-Shaped Funnel

**Figure 4:**
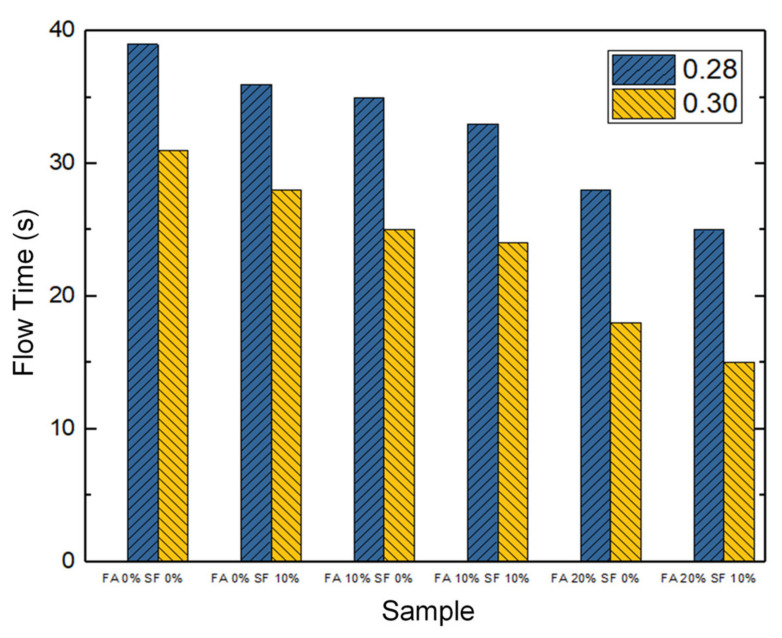
The time taken to flow through the V-shaped funnel by 12 HPC samples with a water/binder (W/B) ratio of 0.28 or 0.30. Note: FA = fly ash; SF = silica fume.

The V-shaped funnel is a device usually applied to measure the viscosity and anti-segregation of self-compacting concrete. The time elapsed for each HPC sample, in its initial form, to flow through the V-shaped funnel, was tested. Figure 4 presents the results.

It is clear that the W/B ratio affected the speed of flow. When the W/B ratio was 0.28, the flow time was between 25 and 39 s, but with a W/B ratio of 0.30, the time was 15 to 31 s. These results suggest that even with a 2% increase in the W/B ratio (with all other conditions being controlled), the influence of higher water content on the viscosity of the concrete sample would be enormous in engineering practice. Further examination revealed that the flow time increased by 20.5% when the W/B ratio was 0.28 compared to 0.30 when neither FA nor SF was added; similarly, it increased by 40% when the mixture was 20% FA and 10% SF (Figure 4).

In addition, at a W/B ratio of 0.28, the sample with 20% FA and 10% SF showed more rapid flow (by 36.89%) compared to that with no FA and SF. This difference in speed became very important when the W/B ratio was 0.30, i.e., the HPC with 20% FA and 10% SF admixture could flow through the V-funnel far faster than the HPC without FA and SF (by 51.61%). Therefore, these results show that adding FA and SF not only enhanced the viscosity and anti-segregation of HPC but also simultaneously improved its cohesion and the formability.

In short, in the case of fresh properties, the measured slump flow of an HPC sample increased with increasing FA and/or SF admixture, and the speed of flow through the V-shaped funnel also increased. Maximal flow (72.3 cm) and maximal speed (15 s) were both observed for the sample with the W/B ratio of 0.30 and 20% FA and 10% SF admixture. However, none of the samples is disqualified because no segregation effect was identified during the experiment, i.e., all of them provided the desired workability.

### 3.2. Hardened Mechanical Properties

Hardened mechanical properties refer to the parametric indices of concrete samples that can be measured during the aging process at certain time points. In this study, we measured the compressive strength (MPa) of each sample, velocity (m/s) of ultrasound pulses travelling through the sample, and surface (sheet) resistivity (kΩ-cm). These tests were made at 1, 7, 28, 56, and 91 days after the samples were produced. Therefore, in addition to being able to trace the aging process for each sample, the results could also be compared across the different sample-proportioning strategies (at any given time). 

#### 3.2.1. Compressive Strength

Compressive strength refers to the maximum compression force that can be vertically applied to a material. The compressive strength of concrete is often the target of quality control. It is inevitably a fundamental measure and mechanical property of HPC materials and is critical for the design of concrete structures. In this study, the compressive strength of each sample was measured at each period of aging. A common definition of compressive strength is as below:

(1) $$\sigma =\frac{P}{A}$$

where *P* is the maximal payload (N), *A* is the area of cylindrical specimen (mm^2^) and σ is exactly the compressive strength (MPa).

**Figure 5:**
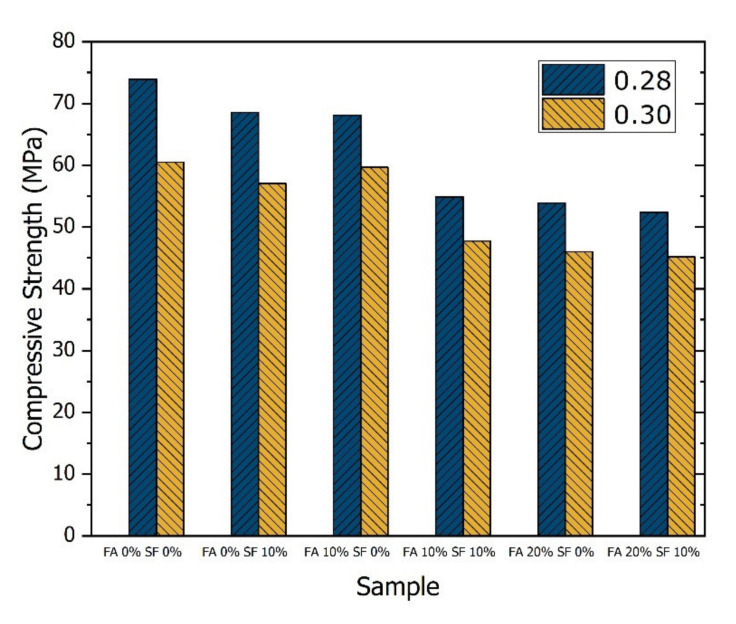
Compressive strength of the 12 HPC samples at day 28 with a water/binder (W/B) ratio of 0.28 or 0.30.

**Figure 6:**
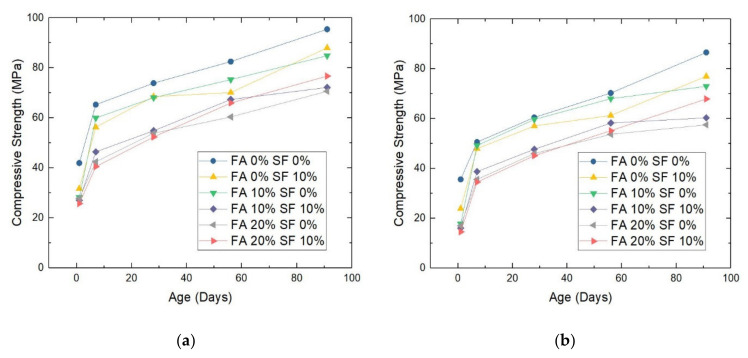
The compressive strength of HPC samples with a water/binder (W/B) ratio of (**a**) 0.28 or (**b**) 0.30. Note: FA = fly ash; SF = silica fume.

The results of this test item could be visualized as histograms once again, e.g., the blended histogram shown in Figure 5. However, Figure 6 shows the results of this test more appropriately. For the six samples produced with a W/B ratio of 0.28, on average, compressive strength increased by 40.8%, 16.7%, 12.1%, and 13.4% after aging for 1–7 days, 7–28 days, 28–56 days, and 56–91 days, respectively. The average compressive strength of the six samples with a W/B ratio of 0.30 showed a more considerable increase of 52%, 19.2%, 13.9%, and 12.5%, respectively, during these periods.

The second insight is the general trend for the compressive strength of a sample to increase with a decreasing W/B ratio. Without any exception, given the same replacement ratio of FA and SF, a sample with a W/B ratio of 0.28 always outperformed its counterpart with a ratio of 0.30. In the samples without any FA and SF, the compressive strength was 9.3–22.5% higher when the W/B ratio was 0.28 than when it was 0.30. The degree to which the W/B = 0.28 sample outperformed its W/B = 0.30 counterpart in compressive strength was 12.5–24.7% for the FA = 0%, SF = 10% combination, 9.7–37.1% for the FA = 10%, SF = 0% combination, 13.1–40.8% for the FA = 10%, SF = 10% combination, 11.1–38.9% for the FA = 20%, SF = 0% combination, and 11.5–43.4% for the FA = 20%, SF = 10% combination.

The third insight is that in this study, the portion of the GGBFS binder was fixed at 30%, and thus the full control (FA 0%, SF 0%) involved the use of 70% Type I cement. Thus, the FA 0%, SF 10% setting involved a reduction of Type I cement to 60%, which further implies a 40% increase in pozzolanic material. Therefore, it is difficult for the FA 0%, SF 10% samples to surpass the compressive strength of FA 0%, SF 0% samples (Figure 5).

Another observational perspective relates to the performance of the full experimental group in comparison with the control group. When neither FA nor SF was used for substitution, for the sample with a W/B ratio of 0.28 and for that with a ratio of 0.30, compressive strength was 41.98 MPa and 35.65 MPa, respectively, on day 1, and 95.43 MPa and 86.59 MPa, respectively, on day 91. When both materials were added for proportioning at 20% FA and 10% SF, i.e., the full experimental group, for W/B = 0.28 and W/B = 0.30 samples, compressive strength was 25.76 MPa and 14.57 MPa, respectively, on day 1, and 76.74 MPa and 69.92 MPa, respectively, on day 91. The extent to which compressive strength decreased in the full experimental group compared to the control early or late in the aging process, depending on the W/B ratio, is undoubtedly the most valuable finding for construction practice. For example, on day 1, compressive strength was reduced by −38.6% in the W/B = 0.28 samples and by −59.12% in the W/B = 0.30 samples under the full experimental condition compared to the control condition.

#### 3.2.2. Ultrasound Pulse Velocity

This test measured the speed at which ultrasound pulses were transmitted through a sample, which was calculated using the thickness of the sample (distance) and the time taken for wave transmission from one edge of the sample to the opposite edge (time). The more solid the internal substances are, the faster the transmission of pulses. This also holds for the tested concrete samples, in that a sample with more interstices has less binding internally; therefore, the speed of the ultrasound waves will be slower because of the detouring effect of the waves. The opposite is that a denser sample has a faster speed (velocity) of ultrasound wave transmission.

**Figure 7:**
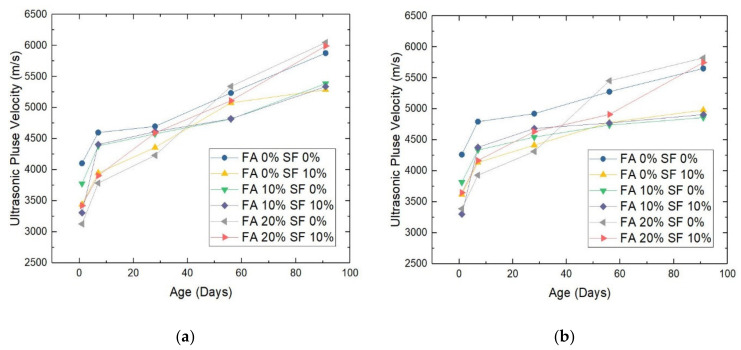
The ultrasound pulse velocity of HPC samples with a water/binder (W/B) ratio of (**a**) 0.28 or (**b**) 0.30. Note: FA = fly ash; SF = silica fume.

For this test, we constructed a cylinder (φ10 cm × 20 cm) using each concrete sample. The results are shown in Figure 7a,b for samples with a W/B ratio of 0.28 and 0.30. Although the trends visualized were similar for the same combination of replacement ratios with different W/B ratios, there are some differences, which could be significant.

On day 1, for the FA = 0%, SF = 0% combination, the velocity of the ultrasound waves penetrating through the W/B = 0.28 sample (see Figure 6a) was lower than that through the W/B = 0.30 sample (see Figure 6b) by −159.67 m/s. This type of difference was shown by all other FA, SF combinations: −178.33 m/s for FA = 0%, SF = 10%; −38.67 m/s for FA = 10%, SF = 0%; −8.02 m/s for FA = 10%, SF = 10%; −261.33 m/s for FA = 20%, SF = 0%; and −225.33 m/s for FA = 20%, SF = 10%. The source data for these figures are provided in Appendix B.

On day 7, the degree to which the velocity of a W/B = 0.28 sample fell behind that of its W/B = 0.30 counterpart was −196.67 m/s, −186.33 m/s, −47.67 m/s, −27.01 m/s, −144.03 m/s, and −262.01 m/s for FA = 0%, SF = 0%; FA = 0%, SF = 10%; FA = 10%, SF = 0%; FA = 10%, SF = 10%; FA = 20%, SF = 0%; and FA = 20%, SF = 10%, respectively. At 28 days, the differences were −225.33 m/s, −56.67 m/s, −32.67 m/s, −72.33 m/s, −82.33 m/s, and −41.02 m/s, respectively. At 56 days, the differences were −42.02 m/s, −297.33 m/s, −76.01 m/s, −46.67 m/s, −112.03 m/s, and −200.67 m/s, respectively. Finally, at 91 days, the differences were −224.67 m/s, −305.67 m/s, −527.01 m/s, −429.33 m/s, −225.03 m/s, and −244.67 m/s, respectively.

A valuable insight from these results is that with increasing sample age, the difference in transmission speed of the ultrasound wave between a W/B = 0.28 sample and its W/B = 0.30 counterpart became increasingly greater.

Moreover, another interesting finding shown in Figure 7 is that at higher ages (i.e., day 56 and 91), the replacement-ratio combination of FA = 20%, SF = 0% began to outperform all other combinations regardless of the W/B ratio. A reason for this is that with the aging of the concrete sample, FA also went on to react with Ca(OH)_2_. Since insoluble C-S-H gels are produced gradually, they not only fill the interstices but also enhance water tightness, which improves the waterproofing capacity of the material. In short, after the entire hydration process, the porosity of the HPC samples became lower due to the addition of FA (and also because of the gels generated with the hydrates), and substance density was ensured. This result provides a guide to the HPC proportioning process.

In addition, in the ultrasound wave test, a general rule is that the wave speed is proportional to the compressive strength of each sample; therefore, the results of these two tests cross-validate each other. The link between them is the density of the HPC material.

#### 3.2.3. Electrical Resistivity

**Figure 8:**
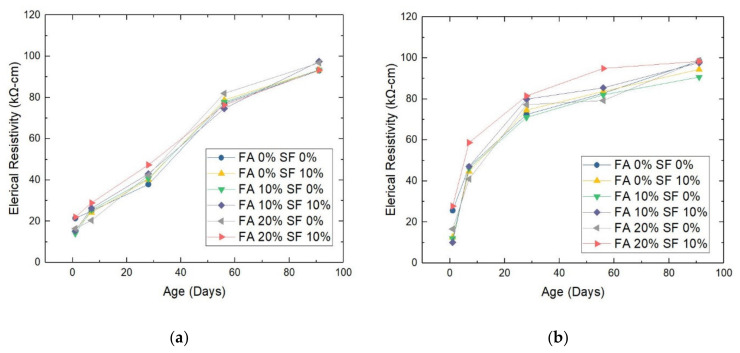
Electrical resistivity of HPC samples with a water/binder (W/B) ratio of (**a**) 0.28 or (**b**) 0.30. Note: FA = fly ash; SF = silica fume.

This test measures the electrical resistance per unit of distance on the surface of a material. A higher resistance of the concrete material signifies that it is more solid; it can thus be used longer. In this study, all samples were tested at the specified days; the results are shown in Figure 8 for the W/B = 0.28 and W/B = 0.30 samples.

The most important insight from this test is the two different general patterns observed for the W/B = 0.28 and W/B = 0.30 sample groups. It is interesting that the trends shown in Figure 8 are not similar for the same FA/SF combinations with different W/B ratios. With the aging of the samples with a W/B ratio of 0.28, their electrical resistivity curves assume a sigmoid shape, while the curves for the individual samples are not different. In contrast, during the aging of the samples with a W/B ratio of 0.30, the shape of the curve is convex for all samples.

The second finding also pertains to the difference in surface resistance between the W/B = 0.28 and W/B = 0.30 samples with the same FA/SF combination. On day 1, without FA and SF (i.e., 0% FA and 0% SF), the surface resistance of the W/B = 0.28 sample (see Figure 6a) was lower by −4.23 kΩ-cm than that of the W/B = 0.30 sample (see Figure 6b). Such a difference was ubiquitous among all other FA/SF combinations: −3.33 kΩ-cm for FA = 0%, SF = 10%; −2.07 kΩ-cm for FA = 10%, SF = 0%; −5.17 kΩ-cm for FA = 10%, SF = 10%; −0.12 kΩ-cm for FA = 20%, SF = 0%; and −5.73 kΩ-cm for FA = 20%, SF = 10%. 

On day 7, the surface resistivity of the W/B = 0.28 samples fell significantly behind that of their W/B = 0.30 counterparts by more than 20 kΩ-cm: −21.92 kΩ-cm, −20.45 kΩ-cm, −20.78 kΩ-cm, −20.78 kΩ-cm, −20.55 kΩ-cm, and −29.83 kΩ-cm, for FA = 0%, SF = 10%; FA = 10%, SF = 0%; FA = 10%, SF = 10%; FA = 20%, SF = 0%; and FA = 20%, SF=10%, respectively. The differences became even greater at 28 days, with all differences more than 30 kΩ-cm: −34.55 kΩ-cm, −34.33 kΩ-cm, −30.33 kΩ-cm, −36.89 kΩ-cm, −34.89 kΩ-cm, and −34.11 kΩ-cm, respectively.

However, at 56 and 91 days, the situation turned around because the surface resistivity values for the two W/B ratios began to converge. This result is the opposite of that for ultrasound wave speed. At 56 days, the differences in surface resistivity between the W/B ratios decreased to −5.66 kΩ-cm, −4.89 kΩ-cm, −4.22 kΩ-cm, −10.70 kΩ-cm, −2.78 kΩ-cm, and 18.55 kΩ-cm, for FA = 0%, SF = 10%; FA = 10%, SF = 0%; FA = 10%, SF = 10%; FA = 20%, SF = 0%; and FA = 20%, SF = 10%, respectively. At 91 days, the differences narrowed further to −5.42 kΩ-cm, −1.11 kΩ-cm, −2.78 kΩ-cm, −0.22 kΩ-cm, −2.03 kΩ-cm, and −5.01 kΩ-cm, respectively.

These results show that the difference between the W/B = 0.28 and W/B = 0.30 samples with the same FA/SF combination is neither linear nor uniform with increasing age of the sample. On day 1, the differences were all small. They were greatly augmented by day 7 and maximal by day 28. On day 56, the differences began to narrow to a certain extent, and by day 91, they became level with those on day 1.

These findings may be due to the fact that at earlier ages, a sample with a W/B ratio of 0.28 may hold less water than one with a ratio of 0.30. This leads to fewer interstices created inside the material during the hydration process. Then, at later ages, such a porous reticular structure will be easily (and effectively) filled up by smaller particles such as FA and SF (indicating a good pozzolanic reaction), ensuring the compactness of the HPC, which eventually provides the desired surface resistivity.

### 3.3. Durability

Following from the discussions in Section 1, durability usually involves environmental conditions, i.e., whether the material can perform in, or is durable in, harsh environments, such as those with harmful substances. However, the degree to which a material can perform under harsh conditions can also be interpreted as how long it can behave as usual under such conditions; therefore, it is a problem of time of use. Because starting an experiment and waiting until a concrete sample fails is infeasible, the method of testing the durability of the sample must be changed.

Following from the explicit definitions in Section 2.1, in this study, to infer the durability of the HPC samples, two tests were conducted, i.e., the anti-sulfate test examining a sample’s ability to endure acid attacks due to sulfates in terms of its corrosive weight loss and the RCPT test examining a sample’s ability to endure negative chloride impacts in terms of its permeability to the accelerated chloride ions.

#### 3.3.1. Anti-Sulfate Capability

**Figure 9:**
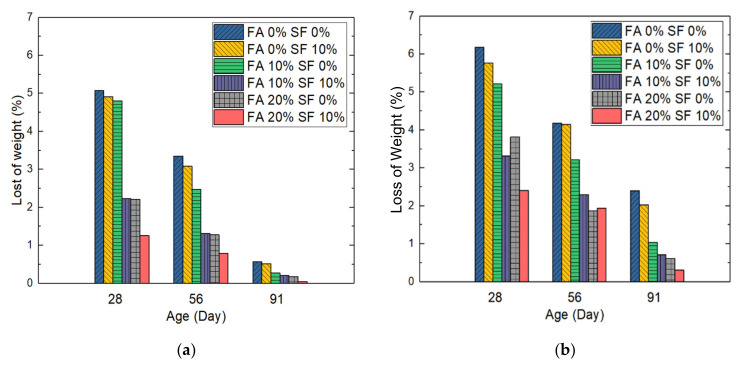
Loss of weight under sulfate-acid attacks of HPC samples with a water/binder (W/B) ratio of (**a**) 0.28 or (**b**) 0.30. Note: FA = fly ash; SF = silica fume.

To evaluate the anti-sulfate capability for the samples, we submerged them in a Na_2_SO_4_ solution; this was repeated for five cycles. We measured the eventual ratio of loss of weight of these samples. The measurements were taken at days 28, 56, and 91 during sample aging because measuring at shorter periods (e.g., 1 or 7 days) was not meaningful for this experiment. The results are visualized in histograms in Figure 9. Based on the figure, two major observations can be made: on the decrease in weight loss during each time window; and on the loss of weight (LoW) values of samples with the same FA/SF combination but with different W/B ratios.

In the case of the first observation, on average, the decrease in the loss of weight of the six W/B = 0.28 samples between day 28 and day 56 was −40.0%, and that between day 56 and day 91 was −86.7%. For the W/B = 0.30 samples, on average, the decrease in loss of weight between day 28 and day 56 was −33.3% and that between day 56 and day 91 was −63.7%. Therefore, the W/B ratio of 0.30 led to a smaller decrease in loss of weight over time.

In the case of the second observation on the loss of weight of samples with the same FA/SF combination but different W/B ratios, the FA = 0%, SF = 0% (i.e., with no FA and SF) sample with a W/B ratio of 0.28 (see Figure 8a) lost less weight (had lower LoW values) than the sample with a W/B ratio of 0.30 (see Figure 8b) at each specific day (−17.9% vs. 76.3%). The FA = 0%, SF = 10% sample with a W/B ratio of 0.28 had a lower LoW value than the sample with a W/B ratio of 0.30 (−14.7% vs. 74.8%). The FA = 10%, SF = 0% sample with a W/B ratio of 0.28 lost less weight than the sample with a W/B ratio of 0.30 (−7.8% vs. −73.8%). The FA = 10%, SF = 10% sample with a W/B ratio of 0.28 lost less weight than the sample with a W/B ratio of 0.30 (−32.8% vs. −70.9%). The FA = 20%, SF = 0% sample with a W/B ratio of 0.28 had a lower loss of weight than the sample with a W/B ratio of 0.30 (−31.6% vs. −71.3%). The FA = 20%, SF = 10% sample with a W/B ratio of 0.28 also had a lower loss of weight than the sample with a W/B of 0.30 (−47.9% vs. −85.1%). 

In the case of the third, samples with a similar portion of pozzolan led to similar results for this test. For example, FA = 20%, SF = 0% samples performed similarly to their FA = 10%, SF = 10% counterparts in relation to sulfate attacks because the same percentage (50%) of pozzolanic materials led to a similar degree of pozzolanic reaction.

In summary, at the three specified days, in all sample pairs with the same FA/SF combination, the sample with the higher W/B ratio (0.30) always lost more weight than its lower-W/B ratio counterpart (0.28). This is demonstrated in Figure 9 in which the two plots have identical scales for comparison of loss of weight.

#### 3.3.2. Rapid Chloride Permeability Test

As discussed previously, it is commonly agreed that among a variety of factors, sulfate acids and chloride ions are the two substances most harmful to the durability of concrete materials. The consensus is that chloride is the most critical factor in corrosion or erosion.

**Figure 10:**
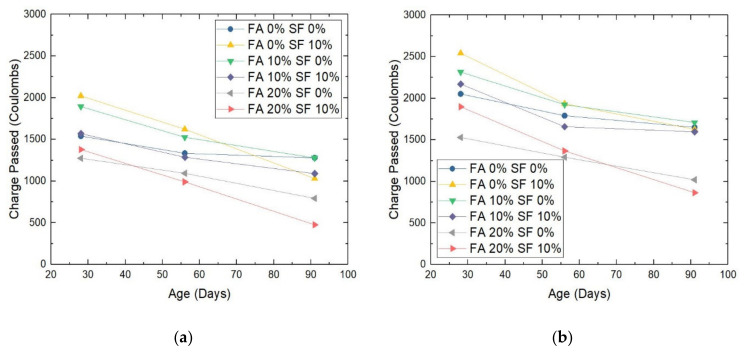
The electric charge passed through HPC samples with a water/binder (W/B) ratio of (**a**) 0.28 or (**b**) 0.30. Note: FA = fly ash; SF = silica fume.

This study performed the RCPT test for all concrete samples in order to understand the degree to which each sample material may block chloride ions, or alternatively, whether or not the material is permeable to chloride ions. In this experiment, with extra voltage applied to excite and accelerate the chloride ions on one edge of the sample, the accumulated electrical charge that successfully passed through the sample body was measured in coulombs (C) at the opposite edge. The results are shown in Figure 10.

Following the logic in Section 3.3.1, three major observations are made. First, for the tested charge passed through samples with the same FA/SF combination but with different W/B ratios, the results are summarized in Table 5.

Second, in the case of the decrease in the measured electric charge during each time window, for the six W/B = 0.28 samples, on average, the decrease in the charge passed between day 28 and day 56 was −18.8%, and the decrease between day 56 and day 91 was −25.2%. For the six W/B = 0.30 samples, on average, the decrease in the charge passed between day 28 and day 56 was −20.1% and that between day 56 and day 91 was −16.1%. In other words, the W/B ratio of 0.30 led to a higher decrease earlier and lower decrease later with increasing age of the samples, but the W/B ratio of 0.28 showed the opposite trend: it led to a lower decrease earlier and a higher decrease later.

Third, unlike the observations in Section 3.3.1, the performance of the FA = 20%, SF = 0% mix was better than (and not on a par with) the performance of the FA = 10%, SF = 10% mix in relation to resistance to chloride ion penetration. This is interesting, but it is reasonable to expect more pore-size refinements with higher FA than higher SF content.

Figure 10 shows that in general, all samples performed well; that is, they became impermeable to rapid chloride ions to some extent by day 91. However, the four samples with 20% FA outperformed the other samples, and among these four, the two samples with 10% SF outperformed the two samples without SF. Therefore, when chloride ions are present in the environment, the use of both FA and SF as substitutes for cement at a replacement ratio of 20% FA and 10% SF (the full experimental group) will be the best choice for HPC. In addition, in this group, the samples with a W/B ratio of 0.28 were the most impermeable to rapid chloride ions; they had a charge of 476.59 C, which is extremely low and justifies the samples being regarded as very durable. An extended discussion is provided in Appendix A.3 for the two reasons for this outcome.

In summary, a higher charge passing through the sample usually means that it is more permeable to chloride ions and is also more porous and vulnerable to other acid attacks, e.g., the sulfates. Consequently, the material is expected to be less durable in practice.

The outcome of the RCPT test reflects the results of the other experiments. In general, it cross-validates the results of the LoW test for sulfate acid attacks. These results provide guidelines for selecting the suitable proportion for concrete mixtures to meet the requirements for undersea construction to establish offshore wind turbines. Besides, surface resistivity, in addition to being a mechanical property, is usually treated as another durability measure. Should this be the case, those results may also cross-validate the results obtained from the two main durability tests in this section.

## 4. Summary

For the results and main findings analyzed in Section 3, Appendix B provides data tables for the experiments for cross-reference. 

As a short summary, in the freshly made HPC samples, the measured slump flow increased with increasing FA or SF, and samples with a W/B ratio of 0.30 always had a higher slump flow than their counterparts with a ratio of 0.28. In addition to these differences, the W/B = 0.30, FA = 20%, SF = 10% combination may have the best workability for engineering practice. In addition, other viscosity and anti-segregation features were measured for each sample by observing the speed of flow through a V-shaped funnel; the results of these tests completely reflect those of the slump flow experiment (i.e., the time, which is the reciprocal of speed, was highly negatively correlated with slump flow distance).

In the case of hardened (non-fresh) mechanical properties, the samples were tested at 1, 7, 28, 56, and 91 days of aging. Overall, the results obtained cross-validated each other; the trends in the results of the three tests were almost identical, with HPC samples with a higher ultrasound pulse velocity usually having higher compressive strength and higher surface resistance. The FA = 20%, SF = 10% combination resulted in superior sheet resistance performance and ultrasound pulse velocity, indicating condensed internal constitution of the material. With a medium value for compressive strength, the W/B = 0.30, FA = 20%, SF = 10% combination should be optimal. In addition, the finding that the six samples with a W/B ratio of 0.28 show a sheet resistivity curve different from that of the six samples with a ratio of 0.30 is interesting and worth further exploration. Moreover, since the experimental results at 91 days are the most meaningful in practice, the degree to which compressive strength was reduced in the full experimental group (FA = 20%, SF = 10%) compared to the control group (FA = 0%, SF = 0%) between day 1 and day 91, depending on different W/B ratios, was analyzed. This analysis also provides valuable knowledge for construction practice.

In the case of durability, in general, the results of two individual tests (the LoW subject to sulfate acid attack and the RCPT) also cross-validated each other. Each durability test was performed at 28, 56, and 91 days of sample aging. The results from the RCPTs revealed that although the W/B = 0.28, FA = 20%, SF = 10% sample was the most impermeable to rapid chloride ions, the W/B = 0.30, FA = 20%, SF = 10% sample was also quite acceptable. Besides, as surface resistivity is often treated as not only a hardened property but also a durability measure, except for the two different resistivity curves observed for W/B = 0.28 and W/B = 0.30 sample groups, the numerical results in Section 3.2.3 also cross-validated those in Section 3.3 for each HPC sample group.

Based on our results, we recommend the production of HPC using the W/B ratio of 0.28 and the combination of 20% FA and 10% SF as a replacement for cement, while keeping the proportion of slag at 30% and of the G-type superplasticizer at 1.5%, in order to ensure the excellent workability, self-compatibility, and overall durability of the material. In addition to this major recommendation, our other results also provide knowledge that is either novel or theory-confirming. As the selected concrete material will be used for grouting underwater foundations of the offshore wind turbines planned to be constructed in the Taiwan Strait, which has harsh natural conditions, the knowledge gained from this study should be both significant and valuable for guiding engineering practice.

Appendix C provides an extensive analysis to understand the relationship between a concrete sample’s compressive strength and the ACP measured using the RCPT. We employed several methods from the data analytics field for this purpose. As a result, we not only identified the relationship between these two specific measures, but also established a bridge between two categories of parameters (i.e., hardened mechanical properties and durability parameters).

Being able to model the relationship between CS and ACP is important, and this outcome should be encouraging. The effort to test concrete materials, e.g., to identify the most suitable proportioning of materials (as we did in this study), can be onerous and time-consuming as many variables need to be considered and numerous experiments need be conducted (e.g., in this study, there were seven different tests). Since the use of ACP to predict CS, or vice versa, is possible, one of the two experimental tests can be omitted.

Therefore, future studies should explore other models to predict one parameter of a concrete sample using another. For example, in this study, there were $C_{2}^{7}$ combinations of paired sample parameters; therefore, there is much to be accomplished in these investigations, but fruitful results can be expected.

## 5. Conclusions

With the increasing importance of offshore wind turbines, a critical issue in the construction of turbines pertains to the HPC material used for grouting undersea foundations. Since these materials must demonstrate better features that are viable under the extremely harsh conditions of the surrounding natural environment, e.g., those present in the Taiwan Bank, the optimal mixture proportioning for producing the most suitable HPC material must be determined.

This study produced and tested 12 types of concrete sample by varying several factors, including the W/B ratio (0.28 vs. 0.30), the replacement ratio for FA (0%, 10%, and 20%) as a substitute for cement, the replacement ratio for SF (0% and 10%), with a fixed proportion of slag of 30% and of G-type superplasticizer of 1.5%. We conducted seven experiments with these samples and gained several interesting insights, either directly from the results or by comparing results, most of which confirmed the theory but some of which went against commonsense. For further details, see the justification based on the experimental results discussed in Section 3 or the summary in Section 4.

A main reason for several experimental results is that due to the smaller grain size of both FA and SF, they are better able to fill the interstices, and consequently, better pozzolanic reactions occur over time during sample aging, i.e., the higher the proportion of these additions, the more condensed the material will be. The results of this study suggest that for HPC material, replacement of cement with 20% FA and 10% SF is recommended for the purpose of constructing foundations to install offshore wind turbines at the desired location in the Taiwan Bank, subject to the abovementioned proportions of slag and superplasticizer. See the inferential discussions in Section 4, which led to this recommendation.

Finally, an effective predictive model was also established with the experimental data to predict the chloride ion permeability of a sample (i.e., its durability) using its compressive strength (i.e., another mechanical property), and vice versa; the model should reduce the effort and time spent on measuring these properties in future experiments because one of the two tests can be omitted. This concept can be generalized to any other pair of parameters (e.g., in the same parametric category or across different categories). This can be one of the main future research directions.

In addition, a boundary condition of this study was that only the best portfolio among the investigated options was investigated and suggested to make the required HPC. Is there a better or optimal option? Answering this question requires changing the preset boundaries in this study, so that further research questions, such as “what if 25% FA and 15% SF are used?”, “what if 35% GGBFS, 20% FA, and 10% SF are used?”, can be asked. Moreover, the expected service life of the foundations of the offshore wind turbines when the different tested mixes are used forms yet another possible direction for advanced knowledge exploration in the future.

## Figures and Tables

**Table 1:** The mixture proportions of the 12 types of concrete samples.

Code	W/B Ratio	Fly Ash (FA) (%)	Silica Fume (SF) (%)	GGBFS (%)	Superplasticizer (%)
W/B = 0.30 FA 0% SF 0%	0.3	0	0	30	1.5
W/B = 0.30 FA 0% SF 10%	0.3	0	10	30	1.5
W/B = 0.30 FA 10% SF 0	0.3	10	0	30	1.5
W/B = 0.30 FA 10% SF 10%	0.3	10	10	30	1.5
W/B = 0.30 FA 20% SF 0%	0.3	20	0	30	1.5
W/B = 0.30 FA 20% SF 10%	0.3	20	10	30	1.5
W/B = 0.28 FA 0% SF 0%	0.28	0	0	30	1.5
W/B = 0.28 FA 0% SF 10%	0.28	0	10	30	1.5
W/B = 0.28 FA 10% SF 0%	0.28	10	0	30	1.5
W/B = 0.28 FA 10% SF 10%	0.28	10	10	30	1.5
W/B = 0.28 FA 20% SF 0%	0.28	20	0	30	1.5
W/B = 0.28 FA 20% SF 10%	0.28	20	10	30	1.5

**Table 2:** Properties of the aggregates used in the concrete samples.

Aggregate (Type)	SSD Specific Gravity (g/cm^3^)	Absorption Capacity (%)	Fineness Modulus
Coarse (9.5 mm)	2.64	1.1	6.43
Coarse (19 mm)	2.65	0.9	7.03
Fine	2.64	1.9	2.79

**Table 3:** Properties of the binding materials used in the concrete samples.

Binding Material	Specific Gravity (g/cm^3^)	Blaine Fineness (cm^2^/g)
Cement	3.15	3310
Fly ash (FA)	2.19	3812
Silica Fume (SF)	2.30	20,000
GGBFS	2.91	4010

**Table 4:** Properties of the superplasticizer.

Type	G
Color	Light brown
pH	7.0 ± 3.0
Specific gravity	1.060 ± 0.03
Effect of water reduction on plasticizing	15–25%

**Table 5:** The electric charge passed through concrete samples of different admixture.

(FA, SF)	A: Charge Passed W/B = 0.28 Sample	B: Charge Passed W/B = 0.30 Sample	% Increase from A to B (W/B from 0.28 to 0.30)
(0%, 0%)	1539.7 C–1278.4 C	2051.9 C–1650.7 C	22.6–25.5%
(0%, 10%)	2021.9 C–1031.7 C	2541.3 C–1626.7 C	16.2–36.6%
(10%, 0%)	1893.7 C–1277.9 C	2351.2 C–1709.2 C	18.2–25.2%
(10%,10%)	1569.9 C–1090.1 C	2170.6 C–1596.9 C	22.4–31.7%
(20%, 0%)	1274.3 C–793.1 C	1528.6 C–1019.6 C	15.3–22.2%
(20%, 10%)	1378.3 C–476.6 C	1897.7 C–864.5 C	27.4–44.9%

**Figure A1:**
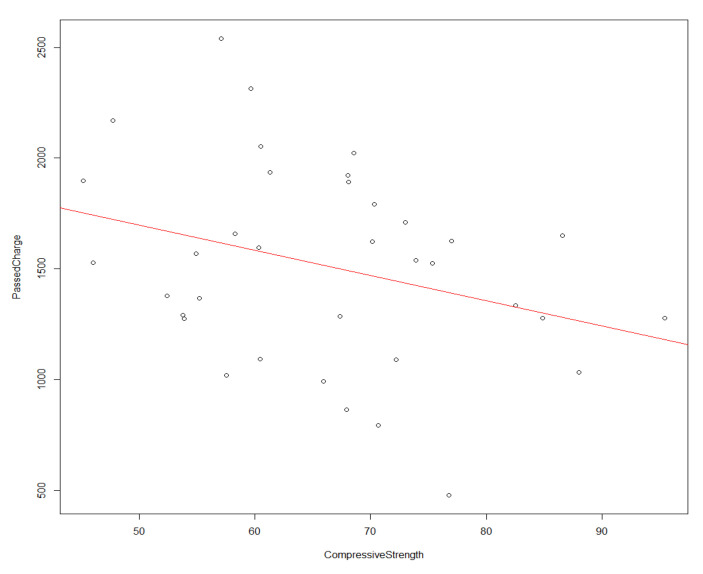
Simple linear regression of compressive strength and accumulated charge passed (small circles: data points (original scale); red line: Equation (2)).

## Data Availability

The study did not report any data availability statement; all data were sourced from real experiments.

## Appendix A

### Appendix A.1

With humankind’s increasing awareness of environmental protection, the pursuit of green power has driven the technologies for RE [38]. In the past decades, a number of RE resources, such as, but not limited to, solar (photovoltaic), wind, hydropower, tidal, and even biomass, were proven to be effective substitutes for traditional energy resources, e.g., thermal and nuclear, which are prone to pollution [39,40].

### Appendix A.2

The climatic (high temperature and high humidity) and geological (due to frequent crustal deformation) features of Taiwan have resulted in its soil being rich in sulfates and crystalline salts. This applies also to the Taiwan Strait, which has a continental shelf topography where the offshore wind turbines are to be constructed; land under water or seawater cannot eliminate acid attacks by sulfates. It is evident that sulfate osmosis occurs in the concrete of inland structures causing serious problems, e.g., the gypsum reaction occurring when the Ca(OH)_2_ in the concrete meets the infiltrated sulfate. This reaction inflates the concrete itself and peels the surface off from the structure. Therefore, anti-sulfate capability is usually a critical durability parameter in this context.

### Appendix A.3

There are two possible reasons for the results obtained for the permeability of HPC samples to rapid chloride ions. The first is the hydration process taking place in FA, SF, and cement to produce Ca(OH)_2_, which optimized the reactional interfaces. The amount of hydrate further enabled the concrete material to lock the chloride ions inside. In other words, the material became more absorptive. The second reason is that the pozzolanic reactions of FA and SF consumed water in the interstices, and the hydrates thus produced filled those interstices hollowed out by water. This made the concrete denser and lowered the porosity of the material, further blocking the routes through which chloride ions would pass.

## Appendix B

This appendix provides several source data sets for the experiments as supplementary information for the discussion. (Table A1, Table A2, Table A3, Table A4 and Table A5).

**Table A1:** Source data for tests on compressive strength (unit: MPa).

CID	W/B	Age (Days)				
		1	7	28	56	91
FA 0% SF 0%	0.28	41.98	65.32	73.92	82.54	95.43
FA 0% SF 10%		31.74	56.31	68.53	70.16	88.04
FA 10% SF 0%		28.21	60.00	68.10	75.33	84.90
FA 10% SF 10%		27.11	46.44	54.88	67.37	72.19
FA 20% SF 0%		27.85	42.47	53.88	60.42	70.66
FA 20% SF 10%		25.76	40.54	52.37	65.95	76.74
FA 0% SF 0%	0.30	35.65	50.59	60.50	70.30	86.59
FA 0% SF 10%		23.89	47.96	57.07	61.28	77.00
FA 10% SF 0%		17.74	49.23	59.67	68.03	73.01
FA 10% SF 10%		16.05	38.73	47.71	58.25	60.35
FA 20% SF 0%		17.03	35.59	46.02	53.74	57.53
FA 20% SF 10%		14.57	34.51	45.17	55.17	67.92

**Table A2:** Source data for tests on ultrasound wave velocity (unit: m/s).

CID	W/B	Age (Days)				
		1	7	28	56	91
FA 0% SF 0%	0.28	4263.67	4794.67	4923.33	5278.00	5653.00
FA 0% SF 10%		3620.33	4135.33	4415.67	4781.00	4981.33
FA 10% SF 0%		3816.00	4336.33	4545.67	4739.33	4859.67
FA 10% SF 10%		3300.67	4380.33	4685.00	4773.00	4908.67
FA 20% SF 0%		3389.33	3930.33	4313.67	5454.33	5823.00
FA 20% SF 10%		3648.67	4167.33	4634.67	4910.67	5748.33
FA 0% SF 0%	0.30	4104.00	4598.00	4698.00	5236.00	5877.67
FA 0% SF 10%		3442.00	3949.00	4359.00	5078.33	5287.00
FA 10% SF 0%		3777.33	4384.00	4578.33	4815.33	5386.67
FA 10% SF 10%		3308.67	4407.33	4612.67	4819.67	5338.00
FA 20% SF 0%		3128.00	3786.33	4231.33	5342.33	6048.00
FA 20% SF 10%		3423.33	3905.33	4593.67	5111.33	5993.00

**Table A3:** Source data for tests on sheet resistance (unit: kΩ-cm).

CID	W/B	Age (Days)				
		1	7	28	56	91
FA 0% SF 0%	0.28	21.43	25.00	37.89	77.11	93.11
FA 0% SF 10%		15.96	24.22	40.22	78.78	93.33
FA 10% SF 0%		14.03	25.44	40.67	77.78	93.44
FA 10% SF 10%		15.27	26.33	43.00	74.67	97.45
FA 20% SF 0%		16.48	20.44	42.33	82.00	96.78
FA 20% SF 10%		22.02	28.89	47.33	76.33	93.33
FA 0% SF 0%	0.30	25.67	46.92	72.44	82.78	98.53
FA 0% SF 10%		12.63	44.67	74.55	83.67	94.45
FA 10% SF 0%		11.96	46.22	71.00	82.00	90.67
FA 10% SF 10%		10.10	47.11	79.89	85.45	97.67
FA 20% SF 0%		16.60	41.00	77.22	79.22	98.78
FA 20% SF 10%		27.76	58.72	81.45	94.89	98.33

**Table A4:** Source data for tests on permeability to sulfate acid (LoW; unit: % of weight).

CID	W/B	Age (Days)		
		28	56	91
FA 0% SF 0%	0.28	5.07	3.35	0.57
FA 0% SF 10%		4.92	3.08	0.51
FA 10% SF 0%		4.81	2.47	0.27
FA 10% SF 10%		2.23	1.32	0.21
FA 20% SF 0%		2.21	1.28	0.18
FA 20% SF 10%		1.25	0.79	0.05
FA 0% SF 0%	0.30	6.18	4.18	2.40
FA 0% SF 10%		5.76	4.15	2.03
FA 10% SF 0%		5.21	3.22	1.04
FA 10% SF 10%		3.31	2.30	0.71
FA 20% SF 0%		3.81	1.87	0.61
FA 20% SF 10%		2.41	1.94	0.30

**Table A5:** Source data for the rapid chloride permeability test (unit: charge passed in coulomb).

CID	W/B	Age (Days)		
		28	56	91
FA 0% SF 0%	0.28	1539.725	1333.971	1278.355
FA 0% SF 10%		2021.985	1622.128	1031.655
FA 10% SF 0%		1893.679	1525.602	1277.911
FA 10% SF 10%		1569.871	1286.292	1090.035
FA 20% SF 0%		1274.315	1093.233	793.1002
FA 20% SF 10%		1378.311	990.3959	476.5927
FA 0% SF 0%	0.30	2051.903	1790.525	1650.654
FA 0% SF 10%		2541.305	1934.883	1626.577
FA 10% SF 0%		2315.195	1922.696	1709.153
FA 10% SF 10%		2170.629	1658.202	1596.957
FA 20% SF 0%		1528.556	1291.338	1019.569
FA 20% SF 10%		1897.666	1366.819	864.4545

## Appendix C

We treated compressive strength (CS) and accumulated charge passed (ACP) as statistical variables. Table A6 is the new data table created with the original results for samples tested at 28, 56, and 91 days.

**Table A6:** Data table for data analysis and modelling.

Sample #	Compressive Strength (MPa)	Accumulated Charge Passed (C)
1	73.92	1539.72
2	82.54	1333.97
3	95.43	1278.35
4	68.53	2021.99
5	70.16	1622.13
6	88.04	1031.65
7	68.10	1893.68
8	75.33	1525.60
9	84.90	1277.91
10	54.88	1569.87
11	67.37	1286.29
12	72.19	1090.04
13	53.88	1274.32
14	60.42	1093.23
15	70.66	793.10
16	52.37	1378.31
17	65.95	990.40
18	76.74	476.59
19	60.50	2051.90
20	70.30	1790.52
21	86.59	1650.65
22	57.07	2541.30
23	61.28	1934.88
24	77.00	1626.58
25	59.67	2315.20
26	68.03	1922.70
27	73.01	1709.15
28	47.71	2170.63
29	58.25	1658.20
30	60.35	1596.96
31	46.02	1528.56
32	53.74	1291.34
33	57.53	1019.57
34	45.17	1897.67
35	55.17	1366.82
36	67.92	864.45

A simple linear regression (SLR) model was established (Godfrey, 1985) [41], with *ACP* as the dependent variable and *CS* as the independent variable:

*ACP* = 2667.231 + (−11.399)*CS* (A1)

Figure A1 shows the SLR of the data provided in Table A6. The Pearson correlation coefficient between these two variables was −0.3118, and the results for the SLR model (from the R platform) are as follows:

Coefficients:

                    Estimate Std. Error t value Pr(>|t|)    

(Intercept)         2267.231    401.425   5.648 2.47e-06 ***

CompressiveStrength  -11.399      5.958  -1.913   0.0642 .  

---

Signif. codes:  0 ‘***’ 0.001 ‘**’ 0.01 ‘*’ 0.05 ‘.’ 0.1 ‘ ’ 1

Residual standard error: 430.2 on 34 degrees of freedom

Multiple R-squared:  0.09721,     Adjusted R-squared:  0.07066 

F-statistic: 3.661 on 1 and 34 DF, *p*-value: 0.06415

The *p*-value (0.06415) of the *F*-statistic, which indicates whether the model is effective, was <0.1, suggesting that the model was relatively weak (Angus et al., 2012) [42]. The regression coefficient, *R*^2^, which indicates the model fit of the data, was too low (0.09721); therefore, the model fit was poor (Akossou and Palm, 2013) [43]. In other words, the established SLR model did not provide a satisfactory explanation of the relationship between the variables; therefore, alternative methods were explored.

Our first approach was to dimensional alternation of the variables without changing the linearity of the SLR model, which is a common method used in social science research [44,45]. Let *X* be the new independent variable after some dimensional alternation of the original *CS* variable and let *Y* be the new dependent variable (after some other dimensional alternation) of the original *ACP* variable, rendering another SLR model, *Y* = α + (β)*X* + ε. We attempted the following setting with and without outlier removal [46]: *X* = *CS*^2^, *Y* = ln(*ACP*). Without outlier removal, the correlation between *X* and *Y* was −0.3118, the *p*-value of the regression model was 0.08949, and the *R*^2^ value was 0.08246. However, with outlier removal, the correlation between *X* and *Y* was −0.2416, the *p*-value was 0.1686, and *R*^2^ was 0.05838, an even worse result. In this approach, we attempted other dimensional alternations, using the square root, square and log (among others) of the variables, and did not obtain better results; therefore, we used a second approach.

The second approach involved clustering the data points into several groups, using the center of each group as representative of the data points in that group [47], and established an SLR model based on these representative data points. This additional process ensured that the subjects of modelling were converged, and the effect of noise was mitigated; consequently, a better model could be expected.

The 36 data points were classified into four clusters by using K-means, an unsupervised machine-learning method [48,49]. These clusters contained 9, 6, 10, and 11 original data points. The centers of these clusters were located at (*CS*, *ACP*) = (59.56222, 2083.3278), (71.14000, 862.6267), (67.80100, 1267.0580), and (67.80091, 1619.8127), respectively. The within-cluster sum of squares (SS) was 789461.6, 447854.9, 183503.6, and 135182.2, respectively. The subtotal of the within-cluster SSs accounted for 88.8% of the total SS. Table A7 provides a summary of the clusters.

**Table A7:** Clusters of the tested samples.

CID	Cluster Center (CS, ACP)	# Data Points	Data Points (Samples) in the Cluster	Within-Cluster Sum of Squares (Variance)
A	(59.56222, 2083.3278)	9	4, 7, 19, 22, 23, 25, 26, 28, 34	789,461.6
B	(71.14000, 862.6267)	6	6, 15, 17, 18, 33, 36	447,854.9
C	(67.80100, 1267.0580)	10	2, 3, 9, 11, 12, 13, 14, 16, 32, 35	183,503.6
D	(67.80091, 1619.8127)	11	1, 5, 8, 10, 20, 21, 24, 27, 29, 30, 31	135,182.2

An SLR model was then established for the (*CS*, *ACP*) cluster centers. The Pearson correlation coefficient for the relationship between the two new variables *CS* and *ACP* was −0.9293, which means that the data were strongly negatively correlated. Therefore, the following model was established:

*ACP* = 7966.72 + (−97.76)*CS* (A2)

The *R*^2^ of the new SLR model was 0.8386. Compared to the previous model, the fit of the data to this model was greatly improved. In addition, although the *p*-value for the model was still <0.10 (0.07071), and it was slightly higher than that for the SLR model fitted using the original data, the final SLR model was acceptable given both the high *R*^2^ and the *p*-value for the fit of the data to the model, and the effectiveness of prediction. We supply a final note here for how we have used *k* = 4 as the number of clusters for K-means. The publication entitled “Learning the k in k-means” [48] stated that “choosing *k* is often an ad hoc decision based on prior knowledge, assumptions, and practical experience”. As such, this study makes the ‘ad hoc decision’ for the value of *k* by reference to the ‘prior knowledge’ that in most application studies of K-means, the suitable range for *k* is {3, 4, 5, 6, 7} (e.g., in: https://uc-r.github.io/kmeans_clustering, accessed on 6 October 2021), with an objective to fulfil the explicit aim of this study (i.e., we pursue an acceptable *R*^2^ value of the predictive model). As using this approach to determine *k* has been supported by other studies in real practice (e.g., in [49]), and since in this study at *k* = 4 the *R*^2^ of the model is acceptable (>0.8), the value of *k* is determined subject to such ‘practical experience’.
